# Hepatoprotective Activity of *Cucumis trigonus *Roxb. Fruitagainst CCl_4_ Inducesd Hepatic Damage in Rats

**Published:** 2011

**Authors:** Kalpana Patil, Shaikh MohammedImtiaz, Anoop Singh, Varsha Bagewadi, Shaikh Gazi

**Affiliations:** a *Department of Pharmacognosy and Phytochemistry. KLES’s College of Pharmacy, Belguam, Karnataka, India.*; b *Department of Pharmaceutical Chemistry. K. T Patil College of Pharmacy, Osmanabad, Maharashtra, India*

**Keywords:** Hepatoprotective, Hepatotoxicity, CCl_4_, *Cucumis trigonus*, Liver

## Abstract

In India, a number of medicinal plants and their formulations are used to cure hepatic disorders in traditional systems of medicine. No systemic study has been done on protective effect of *Cucumis trigonus* Roxb. (Cucurbitaceae) to treat hepatic diseases. Protective action of *C. trigonus* fruit extracts was evaluated in this study in animal model of hepatotoxicity, which was induced by carbon tetrachloride. Forty two healthy female albino Wistar rats weighing between 180 and 200 g were divided in to seven groups of 6. Group 1 was normal control group; Group 2, the hepatotoxic group was given CCl_4_; Group 3 was administered standard drug (Liv-52); Groups 4-7 received pet. ether, chloroform, alcohol and aqueous fruit extract (300 mg/kg) with CCl_4_. The parameters studied were alanine transaminase, aspartate transaminase, alkaline phosphatase and serum bilirubin activities. The hepatoprotective activity was also supported by histopathological studies of liver tissue. Results of the biochemical studies of blood samples of CCl_4_ treated animals showed significant increase in the levels of serum enzyme activities, reflecting the liver injury caused by CCl_4_. Whereas blood samples from the animals treated with chloroform and aqueous fruit extracts showed significant and alcohol extract showed highly significant decrease in the levels of serum markers, indicating the protection of hepatic cells. The results revealed that alcoholic fruit extract of *Cucumis trigonus *could afford highly significant protection against CCl_4_ induced hepatocellular injury.

## Introduction

Liver disease is a worldwide problem. Liver is an organ of paramount importance as it plays an essential role in maintaining the biological equilibrium of vertebrates (1). Additionally, it is the key organ of metabolism and excretion is continuously and variedly exposed to xenobiotics because of its strategic placement in the body. The toxins absorbed from the intestinal tract gain access first to the liver resulting in a variety of liver ailments. Thus liver diseases remain one of the serious health problems (2). Conventional or synthetic drugs used in the treatment of liver diseases are sometimes inadequate and can have serious adverse effects (3-5).

Therefore, many folk remedies from plant origin are evaluated for its possible antioxidant and hepatoprotective effects against different chemical-induced liver damage in experimental animals. CCl_4_-induced hepatotoxicity model is frequently used for the investigation of hepatoprotective effects of drugs and plant extracts. The changes associated with CCl_4_-induced liver damage are similar to that of acute viral hepatitis (6).

*Cucumis trigonus known as ‘ Bitter gourd” is a plant belonging to the Cucurbitaceae family and is indigenous to india, ceylon, malaya, north australia, afghanistan and persia* (7)*. *In Indian traditional system of medicine the fruit pulp of the plant is used as expectorant, liver tonic, stomachic and purgative. The fruit pulp is useful in leprosy, jaundice, diabetes, bronchitis and amentia (8). No systematic study has been done on protective efficacy of *Cucumis trigonus *to treat hepatic diseases. Therefore, the protective action of *Cucumis trigonus *fruit extracts was evaluated in an animal model of hepatotoxicity induced by carbon tetrachloride.

## Experimental

*Plant extract*

The fruits of *Cucumis trigonus *Roxb. were collected in August 2008 from Beed, Maharashtra State, India. The plant was authenticated by Dr Harsha Hegde Research officer, regional medical research centre, belgaum, karnataka, india and the voucher specimen has been deposited in the herbarium of the Department of Pharmacognosy and Phytochemistry, K.L.E.S’s College of Pharmacy, Belgaum, Karnataka, India. The collected fruits were shade dried at room temperature.The two hundred grams of dried powdered fruits of *Cucumis trigonus* were extracted by continuous hot extraction process using soxhlet apparatus with petroleum ether, chloroform and alcohol. The powder was finally macerated with chloroform-water IP (*As per Indian Pharmacopeia*) .The extracts were filtered and concentrated under reduced pressure and low temperature (40^o^C) on a rotary evaporator. 

*Phytochemical analysis*

The extracts of the plant material were screened for various classes of natural products using standard qualitative methods as described by Harborne (9).

*Experimental animals*

Female albino Wistar rats weighing between 180 and 200 g were obtained from animal house, Department of Livestock Production, Government Veterinary College, Hebbal, Bangalore, India. Animals were maintained on a standard laboratory diet. Food and water were given *ad libitum*. They were housed in standard stainless-steel cages at a 12 h cycle of light and dark. Room temperature was kept at 22 ± 2°C and humidity maintained at 50%. All the chemicalsused were of the analytical grade from standard companies.

*Treatment of animals*

Rats were randomly divided into 7 groups with 6 animals in each group. Group 1 served as negative control and was administered a single daily dose of distilled water by oral gavage for seven days. Liver damaged was induced by administration of CCl_4 _(2 mL/kg, IP as 50 : 50 solution in olive oil) on 1^st^, 4th and 7^th^ day to the animal of remaining group. Group 2 received only CCl_4_, group 3 received CCl_4_ and standard reference Liv-52 4 mL/kg. p.o. for 7 days. The drug control groups (4, 5, 6 and 7) were given the plant extracts orally in doses of 300 mg/kg/0.2 mL (in distilled water), respectively, one hour after the administration of carbon tetrachloride, for 7 days (10, 11). Twenty four h after CCl_4_ injection animals were anaesthetized by light ether anaesthesia and blood was collected from the vena cava, and the serum was separated for subsequent use for different enzyme measurements. The rats were then decapitated and the livers were carefully dissected and cleaned of extraneous tissues. Part of the liver tissue was immediately transferred to 10% formalin for histopathological assessments. 

*Assessment of liver damage*

Liver damage was assessed by the estimation of serum activities of AST, ALT, ALP and total bilirubin according to the method of Reitman, Kind and Mally by using commercially available test kits (12-14).

The livers were removed from the animals and the tissues were fixed in 10% formalin for at least 24 h. Then, the paraffin sections were prepared (Automatic tissue processor, Autotechnique) and cut into 5 μm thick sections using a rotary microtom. The sections were then stained with Haematoxylin-Eosin dye and studied for histopathological changes, such as necrosis, fatty changes, ballooning degeneration and lymphocyte infiltration. Histological damages were scored as: Ø, absent; +, mild; ++, moderate; +++, severe; ++++, extremely severe (15).

*Statistical analysis*

Data were analyzed by one-way analysis of variance (ANOVA), followed by the Tuky’s test for individual comparisons using SPSS software and p *≤* 0.01 was regarded as significant. 

## Results

The yield of dried extract was pet. ether (40-60°C) 10.4 (%), chloroform 2.25 (%), alcohol 1.8 (%) and aqueous 11.7 (%.).The alcohol and chloroform extracts were found to be positive for the presence of steroids, triterpenoids, saponins and glycosides. Aqueous extract was found positive for the presence of carbohydrates, steroid and triterpenoids. However pet. ether extract was found positive for the presence of fats and oils.

Administration of CCl_4_ to rats caused a significant elevation in serum activities of AST, ALT, ALP and serum bilirubin after 24 h. Treatment of rats with 300 mg/kg dose of the *Cucumis trigonus *alcoholic, chloroform and aqueous extracts (p.o.) markedly prevented CCl_4_- induced elevation of AST, ALT, ALP and bilirubin. However, 300 mg/kg of the petroleum ether extract did not prevent elevation of the enzymes. Serum bilirubin (total) levels were also significantly enhanced by CCl_4_ treatment but total bilirubin was remarkably reduced by treatment with 300 mg/kg of the alcoholic, chloroform and aqueous extracts (p.o.). Liv-52 with a dose of 4 mL/kg also significantly prevented CCl_4_-induced elevation of serum AST, ALT, ALP and bilirubin activities (Table 1).

**Table 1:** Effect of different extracts of *C. trigonus*onserum activities of AST, ALT, ALP and Bilirubin of CCl_4_ in toxicated rats

**Group **	**Treatment**	**AST**	**ALT**	**ALP**	**Bilirubin**
1.	Control	104.0 ± 0.258	51.17 ± 0.307	118.2 ± 0.307	0.330 ± 0.002
2.	CCl_4_	935.5 ± 0.670^†^	642.5 ± 0.619^†^	198 ± 0.004^†^	1.198 ± 0.004^†^
3.	Liv 52	127.2 ± 0.401^*^	63.00 ± 0.447^*^	127.0 ± 0.447^*^	0.416 ± 0.004^*^
4.	Pet.ether extract	663.7 ± 1.358	205.8 ± 0.909^*^	191.0 ± 0.894^*^	0.886 ± 0.008^*^
5.	Chloroform extract	67.5 ± 0.500^*^	77.50 ± 0.500^*^	142.8 ± 0.477^*^	0.571 ± 0.007^*^
6.	Alcohol extract	141.8 ± 0.401^*^	69.17 ± 0.401^*^	131.7 ± 0.333^*^	0.443 ± 0.006^*^
7.	Aqueous extract	280.5 ± 0.428^*^	87.17 ± 0.542^*^	157.5 ± 0.562^*^	0.670 ± 0.002^*^

Values are mean ± SEM, N = 6, ^†^p ≤ 0.001 vs. normal control, ^*^p ≤ 0.001 vs. CCl_4_ control Units of AST, ALT and ALP activity are U/Land Bilirubin is mg/dL

Histopathological examinations of the liver sections of the rats treated with CCl_4 _showed centrilobular necrosis, fatty changes, congestion and infiltration of lymphocytes around the central veins. Centrilobular necrosis, which is a more severe form of injury, was markedly prevented by treatment 300 mg/kg doses of alcohol, chloroform and aqueous extract but not 300 mg/kg dose of the petroleum ether extract (Table 2). 

**Table 2:** Effect of *C. trigonus* extract on histopathological damages induced by CCl_4_ in rats

**Microscopic observation**	**Control**	**CCl**_4_	**Liv-52**	**Pet.erther **	**Chloroform **	**Alcohol **	**Aqueous **
				**extract**	**extract**	**extract**	**extract**
Fatty changes	+	+++	+	++	+	+	+
Degeneration in hepatic cord	Ø	+++	+	++	+	+	++
Deformation in hepatocytes	Ø	++++	+	+++	+	+	+
Focal necrosis	Ø	Ø	Ø	Ø	+	Ø	+
Centrilobular necrosis	Ø	++++	Ø	+++	Ø	Ø	Ø
Congestion in central vein	Ø	+++	+	++	+	+	+
Congestion in sinusoids	+	+++	+	+++	++	+	+
Infiltration of lymphocytes	Ø	++	+	+	+	+	+

Ø, absent; +, mild; ++, moderate; +++, severe; ++++, extremely severe; rats were injected with determined concentrations of the *C. trigonus *extracts (p.o.) for seven consecutive days before injection of 2 ml/kg CCl_4_ (IP). Histopathological damages were assessed as explained under materials and methods

## Discussion

In Indian system of medicine certain herbs are claimed to provide relief against liver disorders. The claimed therapeutic reputation has to be verified in a scientific manner. In the present study one such drug *Cucumis trigonus *was taken for the study. The chloroform, alcohol and aqueous extract of *Cucumis trigonus* possess significant hepatoprotective activity. However highly significant effect was seen with alcoholic extract against CCl_4_ damage. The petroleum ether extract did not protect rat liver against CCl_4_ damage.

Our investigation on the extracts showed the presence of steroids, triterpenoids and cardiac glycosides in the alcoholic extract. According to these results, it maybe hypothesized that steroids and triterpenoids, which are present in the alcoholic extract, could be considered responsible for the hepatoprotective activity.

CCl_4_ metabolism begins with the trichloromethyl free radical (CCl_3_^•^) by the action of the mixed function of the cytochrome P_450_ oxygenase system. This free radical, which is initially formed as relatively unreactive, reacts very rapidly with oxygen to yield a highly reactive trichloromethyl peroxy radical (CCl3OO^•^). Both radicals are capable of binding to proteins or lipids, or abstracting a hydrogen atom from an unsaturated lipid, thus, initiating lipid peroxidation (16-19). Lipid peroxidation may cause peroxidative tissue damage in inflammation. Therefore, inhibition of the cytochrome P_450_-dependent oxygenase activity could cause a reduction in the level of toxic reactive metabolites and a decrease in tissue injury. On the other hand, an elevation of plasma AST, ALT, ALP and bilirubin activities could be regarded as a sign of damage to the liver cell membrane.

Many compounds known to beneficial against carbon tetrachloride-mediated liver injury exert their protective action by toxin mediated lipid peroxidation either via a decreased production of CCl_4 _derived free radicals or through antioxidant activity of the protective agent themselves (20). 

## Conclusions

In conclusion, the results indicated that under the present experimental conditions, alcoholic extract of *Cucumis trigonus* fruit showedhepatoprotective effects against CCl_4_ induced liver damage in rats.
